# HUMAN RESOURCE MANAGEMENT PRACTICES, ORGANIZATIONAL CULTURE, AND NURSE STAFFING TURNOVER

**DOI:** 10.1093/geroni/igz038.2724

**Published:** 2019-11-08

**Authors:** Robert Weech-Maldonado, Akbar Ghiasi, Ganisher K Davlyatov, Justin C Lord, Kent Rondeau

**Affiliations:** 1 University of Alabama at Birmingham, Birmingham, Alabama, United States; 2 Louisiana State University at Shreveport, Shreveport, United States; 3 University of Alberta, Edmonton, Alberta, Canada

## Abstract

This study examines whether nursing homes’ (NHs) human resource management (HRM) practices and organizational culture are associated with nursing staff turnover. HRM practices are classified into traditional (employment selection tests, formal performance appraisal systems, and realistic job previews); employee-centered (flexible work hours and job sharing); and high involvement (incentive based/merit pay and self-managing teams). Organizational culture consists of four types: clan culture (friendly working environment); adhocracy culture (dynamic/creative working environment); market culture (results-based organization); and hierarchy culture (formalized/structured work environment). This study used facility survey data from approximately 324 NH administrators (30% response rate) from 2017- 2018, merged with secondary data from LTCFocus, Area Health Resource File, and Medicare Cost Reports. The dependent variables consisted of RN, LPN, and CNA turnover rates (% voluntarily quit), while the independent variables comprised HRM practices and type of organizational culture. Control variables consisted of organizational (ownership, chain affiliation, size, occupancy rate, and payer mix) and county-level factors (Medicare Advantage penetration, income, education, unemployment rate, poverty, and competition). Generalized linear model results show that every unit increase in high-involvement HRM practices is associated with a reduction of 6%, 4%, and 2% in RN, LPN, and CNA turnover rates, respectively. Also compared to hierarchical cultures, nursing homes with a clan culture are associated with a reduction of 62%, 49%, and 33% in RN, LPN, and CNA turnover rates, respectively. HRM practices and organizational cultures that promote employee participation, engagement, and empowerment have the potential to reduce nurse staffing turnover rates among underresourced nursing homes.

